# Pesticides Could Alter Amphibian Skin Microbiomes and the Effects of *Batrachochytrium dendrobatidis*

**DOI:** 10.3389/fmicb.2018.00748

**Published:** 2018-04-20

**Authors:** Krista A. McCoy, Ariane L. Peralta

**Affiliations:** Department of Biology, East Carolina University, Greenville, NC, United States

**Keywords:** amphibian, *Batrachochytrium dendrobatidis* (Bd), chytridiomycosis, declines, disease, microbiome, pesticides, pollution

At least 32% of amphibian species are threatened or extinct (Stuart et al., 2004; IUNC, 2017). Amphibians are thought to be especially sensitive to a milieu of stressors because they rely on their skin to regulate fluid balance, ion transport, and respiration. The important role that amphibian skin plays in these critical physiological processes makes them vulnerable to desiccation and environmental pollutants (McCoy and Guillette, 2009). Amphibian skin, also plays a critical role in regulating health by producing antioxidants (Liu et al., 2010), antimicrobial peptides (reviewed in Rollins-Smith et al., 2005, 2011), and by harboring diverse microbial communities that protect against pathogens (Harris et al., 2006). The symbiotic skin bacteria that persist in the presence of antimicrobial mucosal peptides can inhibit pathogen colonization and infection of the skin (Woodhams et al., 2007; Piovia-Scott et al., 2017). Thus, the skin microbiome is an essential part of the amphibian's innate immune system, and changes to the skin microbiome can lead to higher mortality (Harris et al., 2009a).

It is thought that host-mediated microbiome selection can result in disease resistant phenotypes (reviewed in Mueller and Sachs, 2015). The infectious skin disease, chytridiomycosis, caused by the fungal species *Batrachochytrium dendrobatidis* (Bd) is responsible for more than 200 amphibian population declines and extinctions (Skerratt et al., 2007). Importantly, those amphibian populations that successfully persist in the presence of this fungal pathogen include more individuals with (culturable) skin bacterial isolates that produce antifungal compounds compared to amphibian populations that experience major BD-induced declines (Woodhams et al., 2007; Harris et al., 2009a; Rebollare et al., 2016).

Colonization of skin-associated microbes varies over the amphibian life stage, especially before and after metamorphosis (Kueneman et al., 2014, 2016). Changes in microbiome composition over amphibian life stages influences disease suppression. Resident skin bacteria are known to compete for available space and nutrients leading to Bd inhibition and play a critical role in limiting the colonization and establishment of Bd zoospores of various amphibian species (reviewed in Bletz et al., 2013). For example, in Colorado's boreal toads *Anaxyrus boreas* early life stages depended on the skin microbiome to enhance immune function (Kueneman et al., 2016). Specifically, during the tadpole life stage, microbiomes were enriched in Bd-inhibitory bacteria and reduced in fungal taxa (Kueneman et al., 2016). How early microbiome communities influence the structure of later (metamorph and adult) microbiomes and resistance to Bd is unknown, but data presented below suggests that priority effects might control susceptibility. Determining the environmental factors that alter amphibian microbiomes will inform strategies for mitigating the devastating effects of infectious skin diseases such as Bd (Jiménez and Sommer, 2016).

Pollution influences microbial communities across many contexts, and could be influencing amphibian skin microbiomes leaving species more vulnerable to infectious diseases. In fact, microbial communities are typically the first taxa to respond to synthetic chemicals (Lew et al., 2009). For example, polychlorinated biphenyls (PCBs) and heavy metals are known to alter amphibian gut microbiomes (Kohl et al., 2015; Zhang et al., 2016). In fact, pesticides cause significant shifts in the composition of the GI microbiota across diverse taxa from honey bees to humans (Kakumanu et al., 2016; Velmurugan et al., 2017). Pesticides are also known to decrease soil microbial activity, alter microbial metabolic potential, and alter soil bacteria diversity (Lupwayi et al., 2009; Muñoz-Leoz et al., 2013; Jiao et al., 2017). Repeated annual application of the herbicide glyphosate over 4 years reduced beneficial soil organisms (i.e., free-living diazotrophs, arbuscular mycorrhizal fungi, and dark septate endophytes) in a warm-season grassland community (Druille et al., 2016). Pesticides also reduce microbial diversity and alter microbial community structure in aquatic systems (Muturi et al., 2017). In addition to pollutants affecting bacteria, microbes are also capable of metabolizing pollutants which can lead to variation in host responses (reviewed in Claus et al., 2016).

Here, we argue that if pollutants can directly alter gut, soil, and aquatic microbial communities, and microbial communities can alter toxicity, then environmental contaminants could play an important role in altering the amphibian skin microbiome and disease susceptibility (briefly reviewed in Rollins-Smith et al., 2011). Pollutants have contributed to amphibian declines, and agrochemicals are thought to be especially problematic in a number of contexts (Davidson et al., 2002; Hayes et al., 2006; Davidson and Knapp, 2007; McCoy et al., 2008). Pesticides are globally distributed, transported atmospherically, and are deposited and accumulate in areas where amphibian populations have suffered massive declines or extinctions (Daly et al., 2007a,b; Wania et al., 2007). For example, population declines, and extinctions of several California (USA) amphibian species are associated with wind-borne agricultural chemicals (Davidson et al., 2001, 2002; Sparling et al., 2001; Davidson, 2004; Davidson and Knapp, 2007).

Initially, the idea that pesticides were playing a role in amphibian declines seemed unlikely. Many amphibian declines occurred in natural ecosystems that had not experienced obvious human modification and were considered “pristine” environments. However, we now know that many remote ecosystems, such as the artic and relatively isolated montane forests, are contaminated with synthetic pollutants from distant origins (Sonne et al., 2004; Daly et al., 2007a,b; Wania et al., 2007). Soils in some neotropical montane forests in Costa Rica have much higher concentrations of pesticides than what is found elsewhere in the country (Daly et al., 2007a). Some of the pollutants accumulating in remote montane regions of Costa Rica are known to disrupt the endocrine system and can lead to reproductive feminization [e.g., organochlorines (reviewed in Hayes and Hansen, 2017)]. The unusual female-biased sex ratios observed before devastating chytridiomycosis-induced declines that occurred in Costa Rica suggest that endocrine disrupting pesticides in conjunction with skin infectious disease, could have played an important role in the species declines and extinctions occurring in the region (Lips, 1998).

Many pesticides are known immunotoxins and increase host susceptibility to disease (Hayes et al., 2006; Coors et al., 2008), and this link has been known for more than two decades (e.g., reviewed in Banerjee et al., 1996). For example, exposure to the organochlorine DDT suppresses the humoral immune response, and atrazine exposure suppresses thioglycolate-stimulated recruitment of white blood cells and decreases phagocytic activity (Koner et al., 1998; Brodkin et al., 2007). Pollutants can also contribute to host stress and alter host microbiomes resulting in more disease susceptible hosts (reviewed in Alverdy and Luo, 2017).

Although the mechanisms are rarely determined, an increasing number of studies show interactions between pesticides and disease susceptibility. Although, some studies do not find this connection (Gaietto et al., 2014; Wise et al., 2014; Buck et al., 2015; Rumschlag and Boone, 2015), others have argued that these chemicals can facilitate emergence of infectious disease (e.g., Ross, 2002). For example, sublethal exposure of *Rana clamitans* to pesticides increased their susceptibility to trematode infection (Rohr et al., 2013). Some anti-fungal agents (e.g., itraconazole) have been used as therapeutics in hopes of clearing Bd infections (Garner et al., 2009; Berger et al., 2010; Cashins et al., 2013). The herbicide glyphosate and insecticide carbaryl reduce Bd growth in culture, but host-associated Bd growth was not tested (Hanlon and Parris, 2012). The herbicide atrazine and fungicide chlorothalonil were found to inhibit Bd growth in culture, and when associated with tadpoles (McMahon et al., 2013). Although, Bd infections were reduced they were not completely cleared, and atrazine is a reproductive toxicant that feminizes male frogs, and thus will not aid amphibian conservation efforts (McCoy and Guillette, 2009 reviewed in Hayes et al., 2011).

Pesticide exposure can have long lasting effects and influence vulnerability to disease later in life. Frogs that were exposed to atrazine as tadpoles experienced higher mortality when exposed to chytrid fungus post-metamorphosis relative to non-atrazine exposed animals with the same pathogen loads (Rohr et al., 2013), showing that early pesticide exposure influences later disease susceptibility. In another study, tadpoles that were exposed to one of three fungicides along with Bd showed similar Bd loads relative to the no-fungicide control. In contrast, individuals exposed to pesticides as tadpoles and then exposed to Bd as metamorphs (~2 months after fungicide exposure) had significantly greater Bd abundance and Bd-induced mortality than frogs similarly exposed to Bd but with no previous pesticide exposure (Rohr et al., 2017). Importantly, the fungicides used in these studies are all directly toxic to Bd, but paradoxically increased future Bd infections. One hypothesis that could explain the enhanced mortality induced by early pesticide exposure is that toxicants might alter the community shift that occurs during metamorphosis that establishes a healthy skin microbiome making exposed individuals less well protected against future infections. Indeed, Blanchard's Cricket Frog (*Acris blanchardi*) larvae exposed to 2.5 mg/L of the glyphosate containing pesticide Rodeo correlated with distinct skin bacterial communities compared to control Cricket Frogs (Krynak et al., 2017). Additional studies that investigate the effects of pesticide exposure on amphibian skin microbiome form and function are, in our opinion, desperately needed.

Here we argue that pesticides might exacerbate disease progression, transmission, and mortality by altering host-associated microbiomes in ways that enhance successful colonization of pathogenic microorganisms and increase virulence of colonizers. Environmental pollutants can also directly impact soil and aquatic environmental microbial communities (Lupwayi et al., 2009; Muñoz-Leoz et al., 2013; Karimi et al., 2017), which changes the microbial species pool available to colonize amphibian skin microbiomes. For example, microbial community richness and phylogenetic diversity were lowest at a coal ash contaminated site compared to reference sites (Hughey et al., 2016). Although, the skin microbiomes of the frogs from these sites were not compared, it is known that the microbial species pool in the environment are important for maintaining a diverse skin bacterial community (Loudon et al., 2014). It is possible that the coal ash-induced changes in the environmental microbial pool could alter the resident amphibian skin microbiome leaving them more susceptible to pathogens. However, a brief 12 h exposure of adult spring peepers (*Pseudacris crucifer*) to coal ash, which mimics a single night's breeding event, did not induce noticeable changes in skin microbiota (Hughey et al., 2016). The effects of chronic exposure to coal ash, or exposure at earlier life stages on the structure and function of the adult microbiome are still unknown.

Few studies have directly tested how pesticides, or other pollutants, affect the microbiome of amphibian skin or have determined how those alterations scale up to affect colonization by and virulence of pathogens. However, adult frogs that have reduced bacterial diversity as tadpoles have three times more parasitic worms than adults with unmanipulated microbiota as tadpoles. (Knutie et al., 2017). The identity of the pollutant or mixture, dose, and the life history stage in which the animal is exposed will determine the how the chemicals interact with the microbiome, specific disease organisms and host immune system (Jones et al., 2017). For example, skin peptide defenses were significantly reduced in newly metamorphosed foothill yellow-legged frogs (*Rana boylii*) after exposure to carbaryl. However, these changes did not result in altered survival, growth, or antimicrobial defenses in froglets that were also exposed to chytrid (Davidson et al., 2007). Not all pesticides, will induce immunotoxicity or interact with disease organisms (Buck et al., 2012), nor will we always identify effects if we focus on single concentrations of contaminants. Our challenge is to determine the contexts under which environmental contaminants are interacting with disease organisms.

We focus on pesticides as they are globally distributed and are known to induce amphibian population declines, but other types of pollutants affect microbial and amphibian communities. Before we can fully understand the interaction between toxicant exposure, disease, and their combined role in driving amphibian declines, we must understand how pollutants directly affect the amphibian skin (and gut) microbiome, the disease-causing microorganisms, and how those effects scale up to play a critical role in amphibian disease dynamics (Harris et al., 2006, 2009a,b). Pollutant-disease-microbiome interactions are critically understudied aspects of amphibian disease ecology.

## Author contributions

KM and AP contributed to the development of the ideas, writing, and final approval of this manuscript. KM handled incorporating reviewers comments.

### Conflict of interest statement

The authors declare that the research was conducted in the absence of any commercial or financial relationships that could be construed as a potential conflict of interest.
